# Kallikrein-related peptidase 6 regulates epithelial-to-mesenchymal transition and serves as prognostic biomarker for head and neck squamous cell carcinoma patients

**DOI:** 10.1186/s12943-015-0381-6

**Published:** 2015-05-20

**Authors:** Carola H. Schrader, Markus Kolb, Karim Zaoui, Christa Flechtenmacher, Niels Grabe, Klaus-Josef Weber, Thomas Hielscher, Peter K. Plinkert, Jochen Hess

**Affiliations:** Section Experimental and Translational Head and Neck Oncology, Department of Otolaryngology, Head and Neck Surgery, University Hospital Heidelberg, Heidelberg, Germany; Research Group Molecular Mechanisms of Head and Neck Tumors, German Cancer Research Center (DKFZ), Heidelberg, Germany; Institute of Pathology, University Hospital Heidelberg, Heidelberg, Germany; Hamamatsu Tissue Imaging and Analysis Center (TIGA), BIOQUANT, Heidelberg, Germany; Medical Oncology, National Center for Tumor Diseases (NCT), Heidelberg, Germany; Department of Radiation Oncology, University of Heidelberg, Heidelberg, Germany; Division of Biostatistics, German Cancer Research Center (DKFZ), Heidelberg, Germany

**Keywords:** β-catenin, EMT, HNSCC, KLK6, TMA, Vimentin

## Abstract

**Background:**

Dysregulated expression of Kallikrein-related peptidase 6 (KLK6) is a common feature for many human malignancies and numerous studies evaluated KLK6 as a promising biomarker for early diagnosis or unfavorable prognosis. However, the expression of KLK6 in carcinomas derived from mucosal epithelia, including head and neck squamous cell carcinoma (HNSCC), and its mode of action has not been addressed so far.

**Methods:**

Stable clones of human mucosal tumor cell lines were generated with shRNA-mediated silencing or ectopic overexpression to characterize the impact of KLK6 on tumor relevant processes *in vitro*. Tissue microarrays with primary HNSCC samples from a retrospective patient cohort (n = 162) were stained by immunohistochemistry and the correlation between KLK6 staining and survival was addressed by univariate Kaplan-Meier and multivariate Cox proportional hazard model analysis.

**Results:**

KLK6 expression was detected in head and neck tumor cell lines (FaDu, Cal27 and SCC25), but not in HeLa cervix carcinoma cells. Silencing in FaDu cells and ectopic expression in HeLa cells unraveled an inhibitory function of KLK6 on tumor cell proliferation and mobility. FaDu clones with silenced KLK6 expression displayed molecular features resembling epithelial-to-mesenchymal transition, nuclear β-catenin accumulation and higher resistance against irradiation. Low KLK6 protein expression in primary tumors from oropharyngeal and laryngeal SCC patients was significantly correlated with poor progression-free (p = 0.001) and overall survival (p < 0.0005), and served as an independent risk factor for unfavorable clinical outcome.

**Conclusions:**

In summary, detection of low KLK6 expression in primary tumors represents a promising tool to stratify HNSCC patients with high risk for treatment failure. These patients might benefit from restoration of KLK6 expression or pharmacological targeting of signaling pathways implicated in EMT.

**Electronic supplementary material:**

The online version of this article (doi:10.1186/s12943-015-0381-6) contains supplementary material, which is available to authorized users.

## Background

The Kallikrein-related peptidase 6 (KLK6) belongs to a family of 15 secreted serine proteases with trypsin or chymotrypsin-like activity [1]. KLKs are encoded by a cluster of genes located on human chromosome 19q13.3–13.4 [2]. Deregulation of KLK6 expression occurs in neurodegenerative and skin disorders, and is a common event in human cancer (reviewed in [3, 4]). Most of these cancers, such as glioma, ovarian, breast, uterine, pancreatic, colorectal, gastric, skin, urinary bladder, lung and salivary gland tumors, were characterized by a strong increase of KLK6 transcript and protein levels as compared to normal tissues (reviewed in [1, 5, 6]). Consequently, KLK6 represents a promising biomarker of early diagnosis and/or unfavorable prognosis in several human malignancies. Additionally, KLK6 expression was found in tumor adjacent epithelial and stromal cells, and may considerably contribute to the aggressiveness of cancer cells by a paracrine mode of action [7, 8].

Despite this overwhelming evidence pointing to a critical role of KLK6 in neoplastic transformation and malignant progression, the regulation and function of KLK6 under physiological and pathological settings is poorly understood. The regulation of KLK6 expression under pathological conditions is most likely influenced by multiple mechanisms, including gene copy number imbalances [9, 10], exposure to steroid hormones [11, 12], epigenetic events such as gene promoter hyper-methylation [13–15], oncogenic signaling [16–19], and posttranscriptional control by miRNAs [20, 21].

KLK6 can degrade components of the extracellular matrix *in vitro* and participates in cellular processes such as inflammation, receptor activation, and regulation of apoptosis (reviewed in [1, 3, 5]). Moreover, KLK6 is implicated in tissue remodeling and induction of tumor-relevant processes such as proliferation, migration and invasion (reviewed in [1, 3]). However, recent studies questioned the general oncogenic role of KLK6 and stressed the importance to consider its context-dependent, tumor-protective function, as exemplified in breast and renal cancer [14, 22–25].

So far, the expression of KLK6 in head and neck squamous cell carcinoma (HNSCC) and its association with pathological features or the clinical outcome has not been addressed in larger patient cohorts. HNSCC arise from mucosal epithelia lining of the upper aero-digestive tract and represent one of the most common and lethal human cancers worldwide [26, 27]. While tobacco and alcohol consumption remain the major risk factors, more recent findings have established infection by high-risk human papilloma viruses, especially HPV16, as an important cause for a subgroup of HNSCC [28, 29]. Implementation of intensified and multimodal treatment has improved the clinical outcome of HNSCC, but often causes severe toxicity and debilitating long-term impacts on quality of life accompanied with only limited clinical benefit. Accordingly, only 40-50 % of patients with an advanced disease will survive for five years after primary treatment [26], and appropriate therapy of advanced HNSCC still remains a major challenge. Therefore, prognostic biomarkers are urgently needed for better stratification of patients with high risk for treatment failure, and to support the identification of novel drug targets for more efficient and less toxic therapies.

In the current study, we conducted loss-of-function and gain-of-function approaches in mucosal tumor cell lines to investigate the contribution of KLK6 in the regulation of tumor development and malignant progression. We demonstrate that silencing of KLK6 expression promotes tumor cell proliferation, migration and invasion *in vitro*. This phenotype is accompanied by the induction of epithelial-to-mesenchymal transition (EMT), nuclear accumulation of β-catenin and resistance against irradiation. The clinical relevance of our findings is supported by the fact that low KLK6 protein level in primary HNSCCs serves as unfavorable risk factor for progression-free and overall survival.

## Results

### KLK6 regulates tumor cell proliferation

**Fig. 1:**
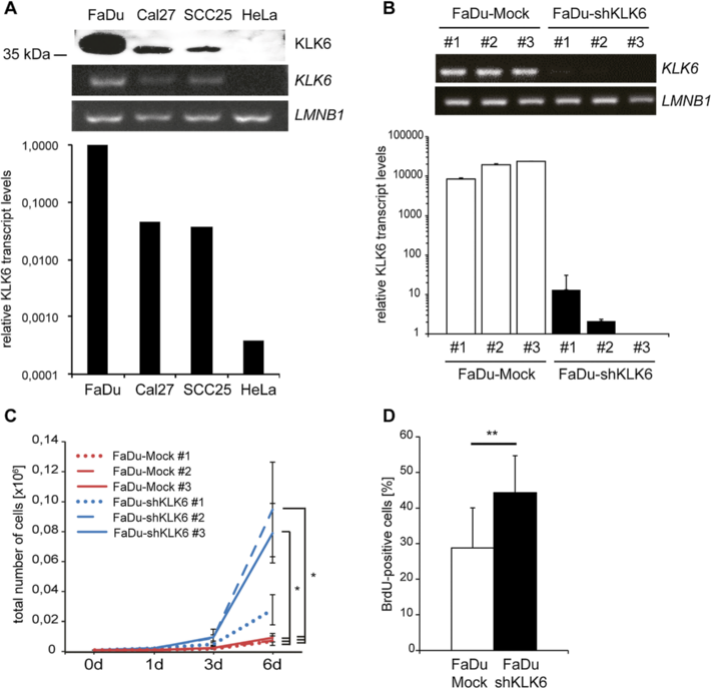
Silencing of KLK6 expression in FaDu cells promotes tumor cell proliferation. (**A**) KLK6 expression in human HNSCC (FaDu, Cal27, SCC25) and HeLa cervix carcinoma cells was monitored on protein level by Western blot analysis with cell culture supernatants (upper panel), and on transcript level by semi-quantitative (middle panels) as well as quantitative RT-PCR (lower graph). Detection of *LMNB1* amplicons served as control for cDNA quality and quantity for semi-quantitative RT-PCR (lower panel), while transcript levels of three independent reference genes (*ACTB, LMNB1, TBP*) were used for quantitative RT-PCR data. (**B**) KLK6 expression in stable FaDu-Mock and FaDu-shKLK6 clones is given by semi-quantitative (upper panel) and quantitative RT-PCR (lower graph) and was determined as described in (**A**). Differences in tumor cell proliferation between stable FaDu-Mock and FaDu-shKLK6 clones was monitored by quantification of cell counts over a time period of six days (**C**) and a BrdU incorporation assay (**D**). The graph represents mean values and standard deviations (SD) of the percentage total of BrdU-positive cells from three independent FaDu-shKLK6 clones and mock controls, respectively

KLK6 expression was monitored on transcript and protein levels in three well-established HNSCC cell lines (FaDu, Cal27 and SCC25) as well as one cervix carcinoma cell line (HeLa). KLK6 transcripts were detected in all three HNSCC cell lines, with highest levels in FaDu cells, while KLK6 expression was almost not detectable in HeLa cells (Fig. 1A). KLK6 expression was also evident on protein level by Western blot analysis, and its presence in the cell culture supernatant demonstrated efficient secretion by HNSCC cell lines under normal growth conditions (Fig. 1A). Following transfection of FaDu cells with control (FaDu-Mock) or pRS-KLK6-shRNA vectors (FaDu-shKLK6), we established stable clones as loss-of-function model to gain a deeper insight into the molecular role of KLK6 on tumor relevant processes. Semi-quantitative and quantitative RT-PCR demonstrated efficient silencing of endogenous KLK6 expression in FaDu-shKLK6 as compared to mock controls, with the highest residual KLK6 transcript level in the FaDu-shKLK6#1 clone (Fig. 1B).

FaDu-shKLK6 clones were characterized by a significant increase in the total number of cells over a time period of six days (Fig. 1C). The degree of the phenotype was associated with the silencing efficiency and less prominent for FaDu-shKLK6#1. A decrease in tumor cell growth was consistent with reduced BrdU incorporation in FaDu-Mock as compared to FaDu-shKLK6 clones (Fig. 1D).

To further support our findings on KLK6-mediated growth inhibition, we transfected KLK6-negative HeLa cells with control (HeLa-Mock) or pcDNA-KLK6-Myc/His plasmids (HeLa-KLK6) to generate stable clones with ectopic KLK6 overexpression (Additional file 1: Fig. S1A-B). Indeed, HeLa-KLK6 clones had a significantly reduced tumor cell growth in comparison to HeLa-Mock controls (Additional file 1: Fig. S1C).

### KLK6 regulates tumor cell morphology, migration and invasion

**Fig. 2:**
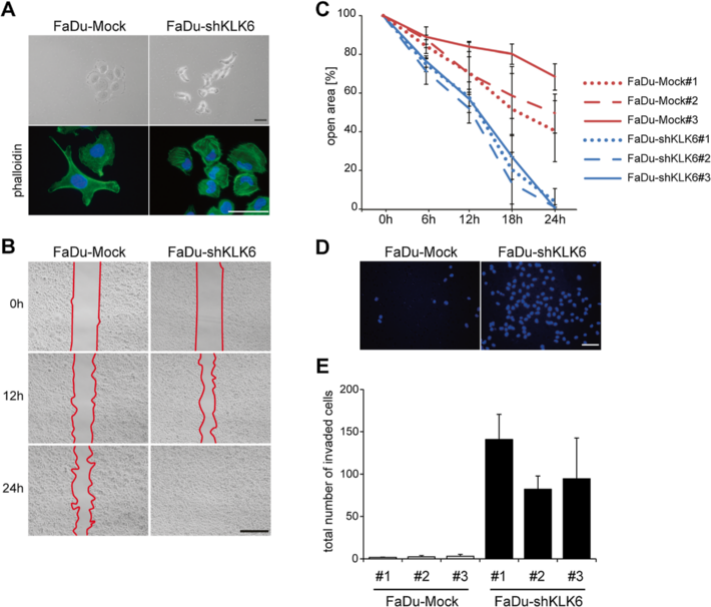
Accelerated tumor cell migration and invasion by KLK6 silencing. (**A**) Representative pictures of FaDu-Mock and FaDu-shKLK6 clones demonstrating a mesenchymal-like morphology (upper panel) after silencing of KLK6 expression accompanied by the presence of stress fibers as determined by phalloidin IF staining (signal in green). Nuclear staining was done with Hoechst 33324 (blue staining); black bar = 50 μm and white bar = 50 μm. (**B**) Representative bright-field pictures of FaDu-Mock and FaDu-shKLK6 clones at the indicated time points of an *in vitro* migration assay. Red lines indicate the border of the migration front. Black bar = 500 μm. (**C**) Differences in tumor cell migration between FaDu-Mock and FaDu-shKLK6 clones is depicted as relative mean values ± SD of three independent experiments. Gap closure was determined by quantification of open areas between two borders at the indicated time points, and the value at the time point 0h was set to 100 %. (**D**) Representative pictures of invasive FaDu-Mock and FaDu-shKLK6 clones in a matrigel-coated Boyden chamber assay after nuclear staining with Hoechst 33324 (blue staining); white bar = 50 μm. (**E**) Quantification of the total amount of invading cells is depicted as mean value ± SD of three independent experiments

Parental FaDu cells and FaDu-Mock controls displayed an epithelial morphology and grew in well-defined cell clusters. In contrast, microscopic inspection of FaDu-shKLK6 clones demonstrated a mesenchymal-like morphology and intracellular stress fibers, easily detectable by phalloidin staining (Fig. 2A). These data suggested a critical impact of KLK6 on tumor cell motility, which was further supported by an accelerated migration rate (Fig. 2B-C), as well as an induced invasion capacity in a matrigel-coated Boyden chamber assay of FaDu-shKLK6 clones as compared to FaDu-Mock controls (Fig. 2E-F).

### KLK6 expression and epithelial-to-mesenchymal transition

**Fig. 3:**
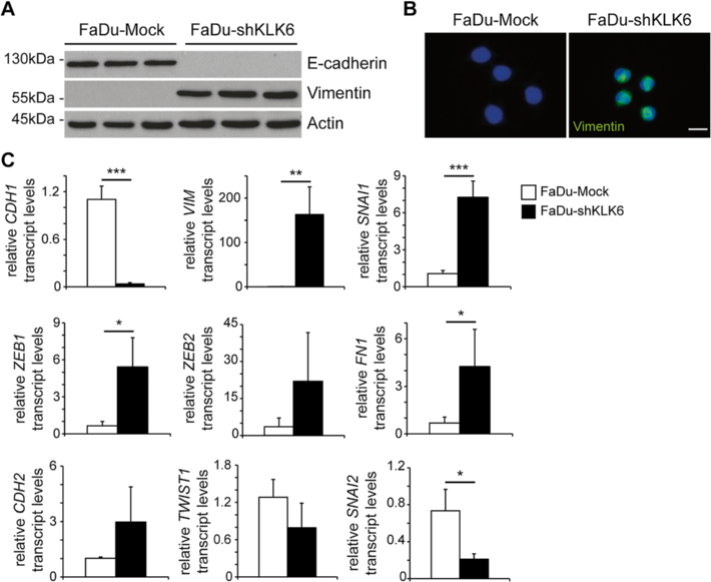
Silencing of KLK6 expression induces an EMT-like phenotype. (**A**) Western blot analysis with whole cell lysate of FaDu-Mock and FaDu-shKLK6 clones demonstrates differences in E-cadherin and Vimentin protein levels. Detection of β-Actin serves as control for quantity and quality of protein lysates. (**B**) Representative pictures of an IF staining confirms Vimentin expression (green signal) in FaDu-shKLK6 but not in of FaDU-Mock clones. Nuclear staining was done with Hoechst 33324 (blue staining); white bar = 20 μm. (**C**) Relative transcript levels of the indicated genes was assessed by quantitative RT-PCR as described above and depicted as mean value + SD of three independent FaDu-Mock and FaDu-shKLK6 clones. *p-value < 0.05, **p-value < 0.005, ***p-value < 0.0005

The mesenchymal-like morphology and accelerated motility of FaDu cells upon loss of KLK6 expression resembled molecular features of EMT. In line with this assumption, Western blot analysis demonstrated an almost complete loss of E-cadherin and prominent induction of Vimentin expression in FaDu-shKLK6 clones as compared to FaDu-Mock controls (Fig. 3A). Induced Vimentin expression in FaDu-shKLK6 clones was also evident by immunofluorescence staining (Fig. 3B). In line with these findings, ectopic KLK6 overexpression in HeLa cells resulted in strongly reduced Vimentin expression (Additional file 1: Fig. S1D).

Next, we investigated the expression of well-established regulators of EMT by quantitative RT-PCR with cDNA derived from FaDu-Mock and FaDu-shKLK6 clones. In addition to significantly induced Vimentin (*VIM*) and reduced E-cadherin (*CDH1*) transcript levels FaDu-shKLK6 clones showed higher transcript levels for SNAIL1 (*SNAI1*), *ZEB1*, *ZEB2*, Fibronectin (*FN1*), and N-cadherin (*CDH2*) (Fig. 3C). In contrast, *TWIST1* and SLUG (*SNAI2*) transcript levels were reduced by KLK6 silencing.

#### Loss of KLK6 expression induces nuclear β-catenin accumulation

**Fig. 4:**
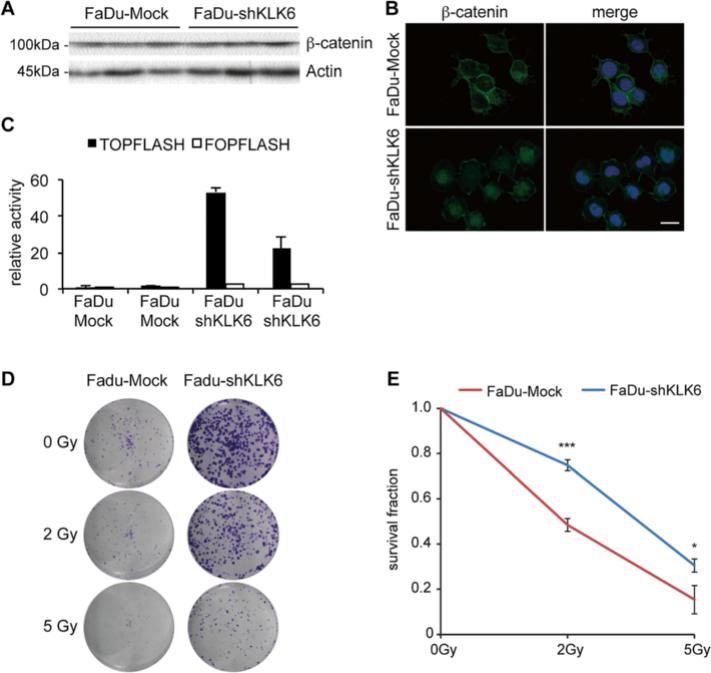
Silencing of KLK6 induces β-catenin signaling and radioresistance (**A**) Western blot analysis with whole cell lysate of FaDu-Mock and FaDu-shKLK6 clones confirm similar β-catenin protein levels. Detection of β-Actin serves as control for quantity and quality of protein lysates. (**B**) Representative pictures of an IF staining demonstrates β-catenin localization (green signal) at the cell membrane of FaDu-Mock controls and its nuclear accumulation in FaDu-shKLK6 clones. Nuclear staining was done with Hoechst 33324 (blue staining); white bar = 20 μm. (**C**) Two independent FaDu-Mock and FaDu-shKLK6 clones were transfected with a TCF-dependent reporter (TOPFLASH) or a mutant control plasmid (FOPFLASH). The graph represents the relative luciferase activity as mean value + SD of three independent experiments. The value of one FOPFLASH-transfected mock control was set to one. (**D**) Representative pictures of a colony forming assay after irradiation with 2 or 5 Gy and of untreated controls (0 Gy). (**E**) The graph represents the relative survival fraction after irradiation at the indicated doses, which is displayed as mean values ± SD and was calculated by the combination of FaDu-Mock#1–3 (red line) and FaDu-shKLK#1-3 (blue line). *p-value < 0.05, ***p-value < 0.0005

Protein-protein interaction networks and functional data indicated that KLK6 might regulate EMT via the TGF-β signaling pathway [14]. However, neither FaDu cells with silenced KLK6 expression nor HeLa cells with ectopic KLK6 expression exhibited major changes in SMAD2/3 phosphorylation (Additional file 2: Figure S2). EMT also has been associated with nuclear accumulation of β-catenin, which is implicated in cell-cell adhesion and a key component of Wnt signaling [30]. Western blot analysis demonstrated no major alteration on the total amount of β-catenin protein in FaDu cells after KLK6 silencing (Fig. 4A). Next, we investigated its intracellular localization by immunofluorescence staining. As expected, prominent staining for β-catenin was detected at the cell membrane of FaDu-Mock controls, while FaDu-shKLK6 clones were characterized by its nuclear accumulation (Fig. 4B). Presence of nuclear β-catenin was accompanied by a strong induction of TCF-dependent transcription as determined by transient transfection of two independent FaDu-Mock and FaDu-shKLK6 clones, respectively, with the TOPFLASH reporter plasmid (Fig. 4C). In summary, activation of Wnt/β-catenin signaling was associated with the EMT phenotype after silencing of KLK6 expression and might contribute to an accelerated self-renewal capacity.

### KLK6 expression and radiosensitivity

An increasing body of experimental evidence supports that an EMT-like phenotype is related to treatment resistance of tumor cells. To investigate whether silencing of KLK6 alters the radiosensitivity of FaDu cells, we performed a colony-forming assay. In the absence of irradiation FaDu-shKLK6 clones developed larger colonies as compared to mock controls, which was consistent with an increase in proliferation (Fig. 4D). In addition, the total number of colonies was higher for FaDu-shKLK6 clones, which might be due to an increased percentage of cells with self-renewal capacity as a consequence of Wnt/β-catenin activity. After irradiation with single doses of 2 and 5 Gy, respectively, the relative survival fraction was significantly higher in FaDu cells with silenced KLK6 expression (Fig. 4E), suggesting a higher radiosensitivity of mock controls.

### KLK6 protein levels in human oropharyngeal and laryngeal SCCs

**Fig. 5:**
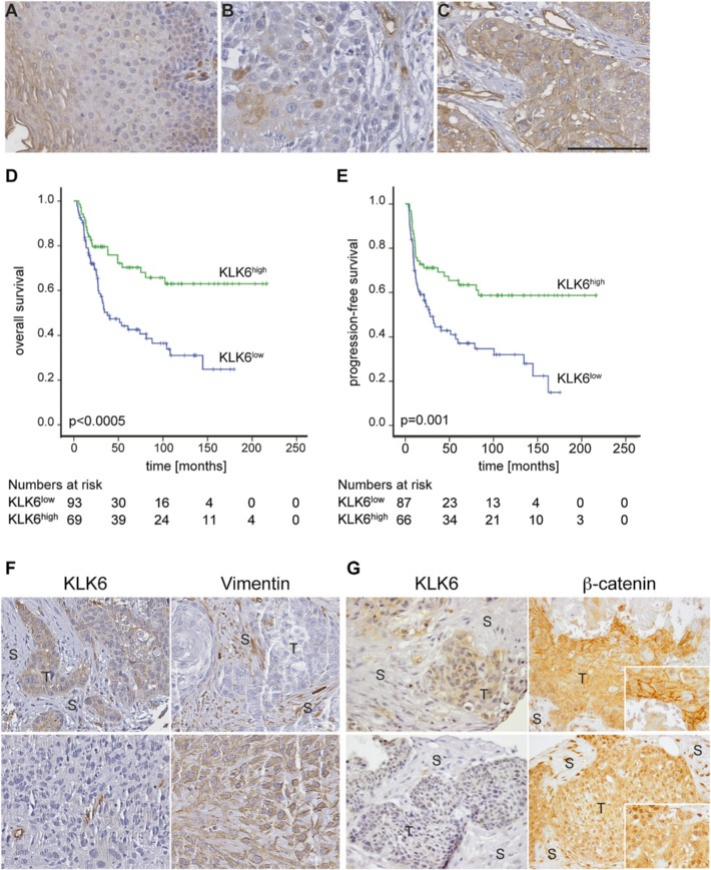
Low KLK6 expression is a risk factor for unfavorable overall and progression-free survival. Representative pictures of an IHC staining on tissue sections of normal mucosa (**A**) and primary HNSCC (**B–C**) demonstrates tumor samples with low (**B**) and high (**C**) KLK6 protein expression (brown signal). Counterstaining with hematoxylin to visualize tissue architecture; black bar = 100 μm. Association between KLK6 protein staining and overall survival (**D**), or progression-free survival (**E**) was assessed by univariate Kaplan-Meier analysis. Green line = subgroup with high KLK6 staining pattern and blue line = subgroup with low KLK6 staining pattern. Representative pictures of IHC staining (brown signal) demonstrate inverse regulation of KLK6 and Vimentin protein expression (**F**), or KLK6-related differences in cellular β-catenin localization (**G**) on serial tumor sections. Inlets represent larger magnifications to demonstrate prominent staining of β-catenin at the membrane or its nuclear accumulation. Counterstaining with hematoxylin to visualize tissue architecture; scale bar = 100 μm. T = tumor and S = stromal tissue

So far, our *in vitro* data provide experimental evidence that loss of KLK6 expression supports proliferation, motility and treatment resistance of cancer cells originating from mucosal epithelia, which is-at least in part-due to the induction of an EMT-like phenotype. To address the clinical relevance of these findings, we determined KLK6 protein levels by IHC staining on tissue microarrays (TMAs) containing tissue samples of two patient cohorts with primary oropharyngeal (OPSCC) or laryngeal squamous cell carcinoma (LSCC). Positive staining was mainly detected in supra-basal keratinocytes of normal mucosa, while a more heterogeneous staining pattern ranging from absent to high KLK6 protein levels in tumor cells was evident in tumor sections (Fig. 5A–C). Staining specificity was further confirmed by IHC staining with an independent anti-KLK6 antibody on serial TMA sections (Additional file 3: Fig. S3). Evaluation of KLK6 expression concerning the relative amount of positive tumor cells and the staining intensity revealed a final expression score for 162 patients, including 115 OPSCCs and 47 LSCCs. The expression score was used to stratify patient subgroups with KLK6^high^ (n = 69) and KLK6^low^ (n = 93) protein levels for further analysis. In the combined patient cohort KLK6 protein expression did not correlate with any of the clinical or pathological features tested, including age, TNM status, clinical stage, pathological grade, and main risk factors, with the exception of gender as females were significantly enriched in the KLK6^low^ patient subgroup (Table 1). Moreover, KLK6 expression was significantly associated with the pathological grade, which was restricted to the LSCC cohort and the HPV status in the OPSCC cohort (Additional file 4: Figure S4).

**Table 1:** Correlation analysis for KLK6 protein expression and clinico-pathological features of the combined HNSCC cohort

		KLK6^low^	KLK6^high^	p-value
Age [years]	<58.62	50	30	0.128
	≥58.62	43	39	
Gender	male	69	60	**0.035**
	female	24	9	
Tumor size	T1/2	39	30	0.509
	T3/4	53	39	
	missing^1^	1		
Lymph node status	N0	27	27	0.129
	N+	65	42	
	missing^1^	1		
Distant metastasis	M0	87	67	0.287
	M+	4	1	
	missing^1^	2	1	
Clinical stage	I/II	16	12	0.581
	III/IV	76	57	
	missing^1^	1		
Pathological grade	G1/2	46	42	0.319
	G3	29	21	
	missing^1^	18	6	
Smoking status	non-smoker	10	9	0.400
	smoker^2^	82	58	
	missing^1^	1	2	
Alcohol consumption	non-drinker	8	9	0.219
	drinker^3^	83	55	
	missing^1^	2	5	

^1^ data missing; ^2^ former and current smokers; ^3^ former and current drinkers. Significant p-values (<0.05) are indicated in bold

To address the question, whether KLK6 expression serves as prognostic biomarker for clinical outcome, we performed Kaplan Meier analysis for progression-free (PFS) and overall survival (OS) of patients in the combined cohort (Fig. 5D–E). The 5-year survival rate for the KLK6^low^ subgroup was 37 % (PFS) and 44 % (OS), respectively, as compared to 65 % (PFS) and 70 % (OS) for the KLK6^high^ subgroup. Accordingly, KLK6^low^ protein staining was significantly associated with reduced PFS (p-value = 0.001) and OS (p-value <0.0005) as compared to patients with KLK6^high^ expression pattern. Kaplan Meier analysis for the individual LSCC and OPSCC patient cohorts revealed similar data (Additional file 5: Table S3) (Additional file 5: Table S4). Next, we performed univariate and multivariate Cox regression analysis to confirm that KLK6^low^ expression serves as an independent risk factor for unfavorable clinical outcome (Table 2) (Additional file 5: Table S5). Finally, we performed IHC staining on serial tumor sections to investigate inverse regulation of KLK6 and Vimentin as well as intracellular accumulation of β-catenin in tumor cells with KLK6^low^ expression levels (Fig. 5F–G).

**Table 2:** Multivariate analysis of overall and progression-free survival for HNSCC patients of the combined cohort

Risk factor		Overall Survival		Progression-Free Survival	
		HR (95 % CI)	p-value	HR (95 % CI)	p-value
KLK6					
	low vs. high^1^	2.793 (1.498–5.209)	**0.001**	2.236 (1.291–3.873)	**0.004**
Age [years]					
	<58.62^1^ vs. ≥58.62	1.588 (0.892–2.825)	0.116	1.359 (0.811–2.277)	0.244
Gender					
	male^1^ vs. female	0.571 (0.268–1.219)	0.148	0.567 (0.281–1.143)	0.112
Clinical stage					
	I/II^1^ vs. III/IV	2.333 (0.892–6.104)	0.084	1.621 (0.724–3.629)	0.240
Pathological grade					
	G1/2^1^ vs. G3	0.811 (0.431–1.527)	0.517	1.135 (0.648–1.987)	0.658
smoking					
	non-smoker^1^ vs. smoker^2^	1.701 (0.602–4.808)	0.317	2.831 (1.013–7.912)	**0.047**
Alcohol consumption					
	non-drinker^1^ vs. drinker^3^	0.911 (0.343–2.418)	0.852	1.056 (0.440–2.535)	0.903

^1^ variable set as reference; ^2^ former and current smokers; ^3^ former and current drinkers. Significant p-values (<0.05) are indicated in bold. HR hazard ratio, CI confidence interval

In summary, these data demonstrate a close association between loss of KLK6 expression and worse clinical outcome of LSCC and OPSCC patients, and strongly support the assumption that KLK6 might serve as a reliable prognostic biomarker to identify HNSCC patients at high risk for treatment failure.

## Discussion

Aberrant expression of KLK6 is a common feature for many human malignancies and numerous studies evaluated KLK6 as a prognostic biomarker (reviewed in [3]). Consistent with most reports, we found high KLK6 expression in primary tumor samples of 42.6 % HNSCC patients. However, while induced KLK6 expression alone or in combination with other KLK family members was associated with poor progression-free and overall survival in colorectal cancer [31, 32], gastric cancer [33, 34], pancreatic ductal adenocarcinoma [35], ovarian cancer [36, 37], lung cancer [38], and intracranial tumors [39, 40], a high KLK6 expression pattern served as favorable prognostic biomarker in our OPSCC and LSCC patient cohorts. These data suggest a context-specific role of KLK6 in regulating the malignant progression and response to therapy, with both tumor promoting and tumor protective functions depending on the cellular origin of the tumor tissue. However, it is worth noting that the tumor promoting function of KLK6 in most human cancers has been deduced from the association of its expression and clinical or pathological features. But only few studies address the underlying molecular mechanisms *in vitro,* and confirmations in preclinical model systems are missing.

So far, only two studies investigated the prognostic value of other KLKs in laryngeal cancer, while to the best of our knowledge no study was published on oropharyngeal SCCs. Both studies reported a remarkable down-regulation of either KLK4 or KLK11 transcript levels in laryngeal cancer as compared to their non-malignant counterparts [41, 42]. Similar to our data on KLK6, patients with KLK11-positive tumors had a favorable prognosis [41], and low KLK4 expression predicted short-term relapse and poor disease-free survival [42]. Finally, down-regulation of KLK13 was reported in oral SCC cell lines and low KLK13 expression in primary oral cancer significantly correlated with regional lymph node metastasis [43, 44].

A tumor protective role of KLK6 was demonstrated in breast cancer, where it was identified originally as a putative tumor suppressor due to its down-regulation during metastasis [22]. Tumor-specific loss of KLK6 expression in breast cancer cells is mediated by epigenetic silencing initiated by hyper-methylation at the proximal promoter [14]. It will be interesting to address whether a similar mode of regulation also occurs in primary HNSCC with low KLK6 expression, and in local recurrence or metastasis that develop in HNSCC patients with treatment failure.

Pampalakis and colleagues demonstrated that restoration of physiological KLK6 levels in breast cancer cell lines reverted the malignant phenotype *in vitro* and *in vivo* [14]. They provided compelling evidence that KLK6 may act as suppressor for tumor progression by promoting a mesenchymal-to-epithelial transition [14]. In line with our findings in HeLa cells, reactivation of KLK6 in breast cancer cells was associated with prominent down-regulation of Vimentin in the absence of a parallel rise in E-cadherin (unpublished data). These data suggest a common inhibitory function of KLK6 on Vimentin expression in breast and mucosal epithelial cells; however, the molecular mechanism remains to be fully elucidated. Moreover, silencing of KLK6 in a HNSCC cell line with prominent KLK6 expression resulted in an EMT-like phenotype with strong up-regulation of Vimentin, complete loss of E-cadherin and deregulation of other well-established molecular markers of EMT. On the cellular level loss of KLK6 was associated with accelerated migration and invasion as well as impaired response to irradiation, well-known characteristics of EMT and most likely responsible for the poor clinical outcome of HNSCC patients with low KLK6 expression. Our data are in contrast to recent findings in other tumor cells lines derived from skin and colon cancer in which KLK6 promotes tumor cell migration and invasion by either E-cadherin ectodomain shedding [45], activation of protease-activated receptors [8] or so far unknown molecular mechanisms [46]. Although the principle nature for these cell type specific differences remains elusive, Pampalakis and colleagues claimed that KLK6 regulates EMT in breast cancer cells via the TGF-β pathway [14, 47]. However, neither FaDu cells with silenced KLK6 expression nor HeLa cells with ectopic overexpression exhibited obvious changes in SMAD2/3 phosphorylation, questioning a major impact of KLK6 on canonical TGF-β signaling, at least in mucosal tumor cells. Intriguingly, our study demonstrated nuclear accumulation of β-catenin and activation of TCF-dependent gene expression upon KLK6 silencing in FaDu cells. Nuclear accumulation of β-catenin was also evident in KLK6^low^ tumor samples of HNSCC patients. Intracellular expression of β-catenin has been reported in the context of invasive growth and metastasis of oral carcinoma cells as well as poor prognosis [48]. β-catenin is a component of the cell-cell adhesion complex and anchored to cadherin-related proteins, which sequester β-catenin at the cell periphery. Consequently, nuclear accumulation of β-catenin might result from down-regulation of E-cadherin during EMT after silencing of KLK6 *in vitro* or loss of E-cadherin expression in KLK6^low^ tumor cells during malignant progression of HNSCCs. In addition, β-catenin is also a key regulator of the Wnt signaling. Wnt/β-catenin is one of the crucial pathways in the maintenance of the self-renewal capacity of stem cells, and its inhibition is a promising approach to target cancer stem cells from HNSCC [49, 50]. In contrast to our data, KLK6-induced nuclear translocation of β-catenin was demonstrated in mouse keratinocytes and human lung cancer cell lines *in vitro* [45, 51], further supporting a diverse and context dependent function of KLK6 in malignant progression.

## Conclusion

**Fig. 6:**
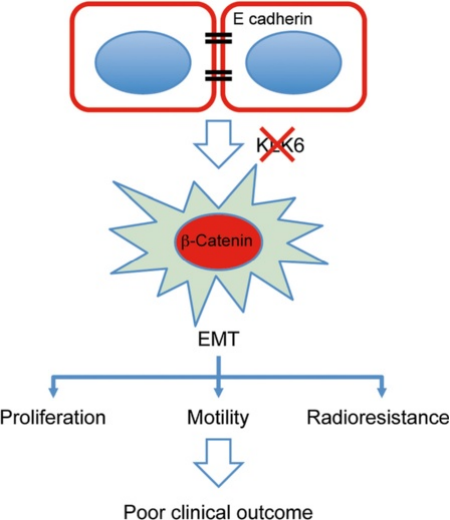
Summary of alterations in molecular, cellular and functional features of HNSCC cells after loss of KLK6 expression. Silencing of KLK6 expression induces an EMT-like phenotype characterized by loss of E-cadherin and gain of Vimentin expression (indicated in green) as well as nuclear accumulation of β-catenin (indicated in red). As a consequence, KLK6-negative tumor cells exhibit increased proliferation, motility and radioresistance, which is in line with the poor clinical outcome of HNSCC patients with low KLK6 expression

In summary, our data demonstrate for the first time that low KLK6 expression serves as unfavorable risk factor for progression-free and overall survival of HNSCC patients, and we provide experimental evidence that KLK6 is a key regulator of tumor cell migration, invasion and response to radiotherapy by modulating EMT and β-catenin signaling (Fig. 6). However, it remains a major challenge for the future to unravel relevant direct targets for KLK6 proteolysis and affected signaling networks that causally contribute to the observed phenotype in mucosal but also breast cancer cells. Furthermore, it will be worth to establish and investigate appropriate pre-clinical models in order to proof the concept, whether HNSCC patients with low KLK6 expression might benefit from pharmacological restoration of KLK6 expression or specific targeting of EMT-related pathways, such as Wnt/β-catenin signaling. This could pave the way for biomarker-driven clinical trials for HNSCC but also other tumor entities in the near future.

## Methods

### Cell culture experiments

Human HNSCC cell lines (FaDu, Cal27 and SCC25 purchased from ATCC) and the cervix carcinoma cell line HeLa were maintained in Dulbecco’s Modified Eagle’s Medium (Sigma, Germany) supplemented with 10 % fetal bovine serum (Sigma, Germany), 2 mM L-Glutamine (Sigma, Germany) and 50 μg/ml Penicillin-Streptomycin (Sigma, Germany) in a humidified atmosphere of 6-8 % CO_2_ at 37 °C. Authentication of all cell lines was confirmed by the Multiplex Human Cell Line Authentication Test (Multiplexion, Germany).

FaDu cells were transfected with either a control pRS vector encoding a non-effective Hush 29-mer scrambled shRNA cassette (TR30012, OriGene Technologies, USA) or a pRS-shKLK6 plasmid (TR316673, OriGene Technologies, USA) using FuGene HD Transfection Reagent (Promega, Germany) according to the manufacturer’s instruction. Following selection with 1.5 μg/ml Puromycin (Gibco life technologies, Germany) for one week, stable clones were isolated and KLK6 silencing was confirmed on RNA level. HeLa cells were transfected with either the pcDNA3.1-Myc/His control or pcDNA3.1-KLK6-Myc/His expression plasmids using FuGene HD Transfection Reagent (Promega, Germany) according to the manufacturer’s instruction. Cloning of pcDNA-KLK6-Myc/His plasmid was described previously [45]. Following selection with 0.4 mg/ml Neomycin (Calbiochem Merck, Germany) for two weeks, stable single clones were isolated and ectopic KLK6 expression was confirmed on RNA and protein level.

### Tumor cell growth and proliferation

1,000 cells per well were seeded in 12-well plates. At the indicated time points after seeding, cells were trypsinized, stained with trypan blue and total number of living cells was determined with the help of a Neubauer counting chamber. For the BrdU cell proliferation assay, 20,000 cells per well were seeded on glass coverslips in 12-well plates. 48 hours after seeding, cells were treated for 30 minutes with 1 mM BrdU, washed three times with 1xPBS for five minutes, and fixed for seven minutes with cold methanol (-20 °C). After intense washing with 1xPBS, fixed cells were treated for 30 minutes at 37 °C with 2M HCl, followed by incubation in borate buffer for 10 minutes. After intense washing with 1xPBS, samples were incubated for 30 minutes at RT with 10 % goat serum in 0,1 % Triton-X-100 in PBS. Next, samples were incubated overnight and at 4 °C with the Alexa555-conjugated BrdU-antibody (diluted 1:100 in PBS). After intense washing with 1xPBS stained cells were incubated with Hoechst 33342 (Calbiochem Merck, Germany) for five minutes and mounted with Mowiol.

### Phalloidin and immunofluorescence staining

20,000 cells per well were seeded on glass coverslips in a 12-well plate. 48 hours after seeding, cells were fixed with 4 % PFA for 15 minutes, treated for 30 minutes with 0.5 % Triton-X100 in PBS, and incubated for 30 minutes with 0.2 % Tween 20/1 % BSA in TBS. Following incubation for 1–2 hours at RT with the first antibody diluted in 0.2 % Tween/1 %BSA in TBS, the secondary antibody diluted in 0.2 % Tween/1 % BSA in TBS was added. After incubation for 30–60 minutes at RT, nuclei were visualized by incubation of cells with Hoechst 33342 (Calbiochem Merck, Germany) and cytoskeleton rearrangement was visualized by incubation with Phalloidin-Alexa Fluor 488 (Invitrogen, Germany) as described previously [45]. Finally coverslips were mounted with Mowiol. Reagents and antibodies used are listed in Additional file 5: Table S2.

### Migration and invasion assay

To assess tumor cell migration, 21,000 cells were seeded into each chamber of sterile culture inserts (ibidi, Germany), which were placed in 6-well plates. 12 hours after seeding, cells were treated for one hour with 10 μg/ml Mitomycin C (Sigma, Germany) in the incubator and insert were removed. Pictures were taken with the Nikon Eclipse Ti microscope using the Nikon Imaging Software NIS-Elements 3.20.02 at the indicated time points and analyzed with the software tool ImageJ to quantify the kinetic of gap closure over time.

To determine tumor cell invasion, FaDu-shKLK6 and FaDu-Mock cells were treated for one hour with 10 μg/ml Mitomycin C (Sigma, Germany), trypsinized and 100,000 cells were seeded in matrigel-coated Boyden chamber inserts (BD Bioscience, Germany), which were placed in 12-well plates. After 48 hours, non-invading cells were removed using cotton swaps and remaining cells were fixed for ten minutes in 4 % PFA and stained with Hoechst 33342 (Calbiochem Merck, Germany). Following mounting with Mowiol, five representative pictures were taken with the Nikon Eclipse Ti microscope using the Nikon Imaging Software NIS-Elements 3.20.02, and the amount of invaded cells was counted with the software tool ImageJ.

### Colony forming assay

To determine the response to irradiation, 100, 300 and 1,000 cells were seeded per well in 6-well plates and irradiated at a dose of 2 or 5 gray (Gy) using X-RAD 320 (Precision X-Ray, North Branford, CT USA) or kept untreated as controls. After 9 days in culture, clones were stained with crystal violet and total number of colonies was quantified as described in [52]. The survival fraction was computed according to [53].

### RNA extraction, cDNA synthesis and RT-PCR analysis

Total RNA from tumor cell lines was isolated with the RNeasy Mini Kit (Qiagen, Germany) following the manufacturer’s instruction. DNA digestion was performed with RNase-free DNAse Set (Qiagen, Germany). Quantity and quality of isolated RNA was determined with the help of the Nanodrop Spectrophotometer ND-1000 (peqlab, Germany). For cDNA synthesis 5 μg total RNA were diluted in 35 μl nuclease-free H_2_O. 0.5 μl Oligo (dT) primers were added and incubated for five minutes at 70 °C. A mixture of 10 μl 5xRevertAid Buffer, 2 μl 25 mM dNTP mix, 2 μl Ribolock RNase Inhibitor and 0.5 μl RevertAid Reverse Transcriptase (all from Fermentas, Germany) was added per reaction and incubated for one hour at 42 °C. For semi-quantitative RT-PCR a ready-to-use mix (Red Load Taq Master, Jena Bioscience, Germany) was used according to manufacturer’s instructions. Amplicons were separated by 1 % agarose gel electrophoresis and visualized with GelRed (Biotium, USA) using the UV documentation system for agarose gel (peqlab, Germany). Quantitative RT-PCR was performed as described previously [54]. As reference genes transcript levels of *ACTB*, *LMNB1* and *TBP* were quantified. Specific annealing temperatures and sequences of all primers are listed in Additional file 5: Table S1. The cycle of threshold (CT) of the gene of interest was standardized to the CT values of the reference genes (ACTB, LMNB and TBP) using the ∆∆CT method. For each primer pair, primer efficiency was tested by a dilution series from 0.01 to 100 ng of a cDNA mix of all samples. A primer efficiency of 1.8 to 2.0 was accepted for further analysis.

### Western blot analysis

Western blot analysis was performed with whole cell lysates or enriched protein fractions from cell culture supernatants as described previously [45]. Antibodies and dilutions that were used for Western blot analysis are listed in Additional file 5: Table S2.

### Luciferase reporter assay

30,000 FaDu-Mock and FaDu-shKLK6 cells were seeded in 24-well plates and transfected with either 0.3 μg TOPFLASH or 0.3 μg FOPFLASH (upstate biotechnology, USA) using FuGene HD Transfection Reagent (Promega, Germany) according to the manufacturer’s instruction. As reference for transfection efficiency all cells were co-transfected with 0.3 μg pTK-RL (Promega, Germany). Firefly and Renilla luciferase activity was quantified with a Sirius Luminometer (Berthold Detection Systems, Germany) using the Dual-Luciferase Reporter Assay System (Promega, Germany) and according to the manufacturer’s instruction.

### *Patient material*

The retrospective study cohort included laryngeal (LSCC) and oropharyngeal squamous cell carcinoma (OPSCC) patients, who were treated at the University Hospital Heidelberg between 1990 and 2008. Paraffin-embedded tissue specimens were provided by the tissue bank of the National Center for Tumor Disease (Institute of Pathology, University Hospital Heidelberg) after approval by the local institutional review board (ethic vote: 206/2005). The study was performed according to the ethical standards of the Declaration of Helsinki. For all tumor samples, clinical and follow-up data were available from the Department of Otolaryngology, Head and Neck Surgery at the University Hospital Heidelberg.

### Tissue microarray and IHC staining

TMAs were generated as previously described [55]. Briefly, hematoxylin and eosin stained sections were cut from each donor block and a certified pathologist defined representative tumor regions. Small tissue cylinders with a diameter of 0.6 mm were taken from selected areas of each donor block using a tissue chip micro-arrayer (Beecher Instruments, Silver Spring, MD, USA) and transferred to a recipient paraffin block. The recipient paraffin block was cut in 2 μm paraffin sections using standard techniques. Immunohistochemistry was conducted with an anti-KLK6 antibody (R&D Systems, Germany) listed in Additional file 5: Table S2 using the TSA Amplification Kit (Perkin Elmer, Germany) according to the manufacturer’s instruction. Counterstaining was done with hematoxylin to visualize tissue integrity.

TMAs were scanned using the Nanozoomer HT Scan System (Hamamatsu Photonics, Japan) capable of scanning whole slides. The Nanozoomer HT Scan System generates digital images of full tissue sections, allowing large-scale histologic evaluation across the complete section. The slides were scanned at a 40-fold magnification (0.23 μm/pixel). Three experienced observers analyzed scanned slides without prior knowledge on the expected results using the NDP Viewer software (version 1.1.27). The semi-quantitative analysis was performed according to two independent categories: first, the number of stained tumor cells (ranging from 1 to 4: score 1 = no positive cells, score 2 = less than 33 %, score 3 = between 34 and 66 %, and score 4 = more than 66 % positive cells), and second, the staining intensity (ranging from 1 to 4: score 1 = no staining, score 2 = weak staining, score 3 = moderate staining, and score 4 = high staining). Subsequently, both scores were multiplied resulting in the final expression score ranging from 1–16, and patient subgroups were arranged according to KLK6^high^ or KLK6^low^ expression.

### *Statistical analysis*

Statistical analysis was done using SPSS (version 21) and SAS (version 9.3) statistics software. Differences between groups were assessed using Chi square test. Overall survival (OS) was calculated as the time from the date of primary tumor diagnosis to the date of cancer-related death within the follow-up interval (events). Survival times of patients who were alive or were dead due to causes other than cancer were censored. Progression-free survival (PFS) was calculated from the date of primary tumor diagnosis to the date of the first local recurrence, lymph node or distant metastasis, second primary carcinoma, or date of cancer-related death within the follow-up period (events). Patients without progression (no event) or cancer-unrelated death were censored. The method of Kaplan-Meier was used to estimate survival distributions. Differences between groups were determined by log-rank tests. A multivariate Cox proportional hazard model was used to assess the association between KLK6 expression scores and overall and progression-free survival of cancer patients from both cohorts, together with the covariates age (continuous variable), clinical stage (IV vs. I–III), gender (female vs. male), TNM, grading, alcohol and tobacco consumption (never vs. former and current consumer). The validity of the proportional hazards assumption was tested with the Supreme Test for proportional hazards assumption and was met for all covariates. In all statistical tests, a p-value of 0.05 or below was considered as statistically significant.

## Additional files

Additional files 1: Figure S1. Decreased tumor cell growth and Vimentin expression in HeLa cells with ectopic KLK6 expression. KLK6 transgene expression in stable HeLa clones was confirmed on transcript level by semi-quantitative RT-PCR (**A**) and on protein level by Western blot analysis (**B**). Detection of *LMNB1* amplicons served as control for cDNA quality and quantity for semi-quantitative RT-PCR, while detection of β-Actin served as control for quantity and quality of protein lysates. (**C**) Differences in tumor cell growth between stable HeLa-Mock and HeLa-KLK6 clones were monitored by quantification of cell counts over a time period of one week. (**D**) Western blot analysis with whole cell lysate of HeLa-Mock and HeLa-KLK6 clones demonstrates reduced Vimentin protein levels in the presence of ectopic KLK6 expression. Additional files 2: Figure S2. KLK6 expression has no impact on canonical TGFβ signaling. Western blot analysis with whole cell lysate of FaDu-Mock and FaDu-shKLK6 clones (**A**) or HeLa-Mock and HeLa-KLK6 clones (**B**) were conducted with anti-pSMAD2/3 or anti-SMAD2 antibodies. Detection of β-Actin served as control for quantity and quality of protein lysates. Additional files 3: Figure S3. Confirmation of staining specificity by IHC staining with two independent anti-KLK6 antibodies. Representative pictures of an IHC staining (brown signal) with two independent anti-KLK6 antibodies (**A** and **C**, AF2008 from R&D; **B** and **D**, sc20264 from Santa Cruz) on serial TMA sections with primary HNSCC samples. Counterstaining with hematoxylin to visualize tissue architecture; black bar = 100 μm. Additional files 4: Figure S4. KLK6 protein staining serves as risk factor for unfavorable progression-free and overall survival of LSCC and OPSCC patients. Association between KLK6 protein staining and overall survival (**A** and **C**) or progression-free survival (**B** and **D**) was assessed by univariate Kaplan-Meier analysis for the LSCC (**A–B**) and OPSCC patient cohort (**C–D**). Green line = subgroup with high KLK6 staining pattern and blue line = subgroup with low KLK6 staining pattern. Additional files 5: Table S1. List of primer sequences for RT-PCR analysis**Table S2.** List of primary (**A**) and secondary antibodies (**B**) **Table S3.** Correlation analysis for KLK6 protein expression and clinical and pathological features of the LSCC cohort. **Table S4.** Correlation analysis for KLK6 protein expression and clinical and pathological features of the OPSCC cohort. **Table S5.** Univariate Cox regression analysis of overall and progression-free survival for HNSCC patients of the combined cohort.
