# Supplementary figures and images for: Kallikrein-related peptidase 6 regulates epithelial-to-mesenchymal transition and serves as prognostic biomarker for head and neck squamous cell carcinoma patients
Source: Mol Cancer. 2015 May 20;14:107. doi: 10.1186/s12943-015-0381-6 (PMC4437453; doi:10.1186/s12943-015-0381-6)

**A**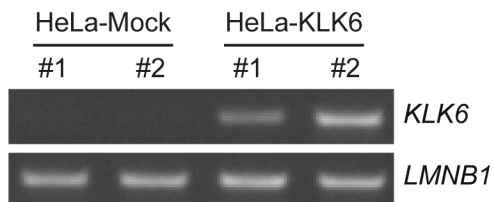**B**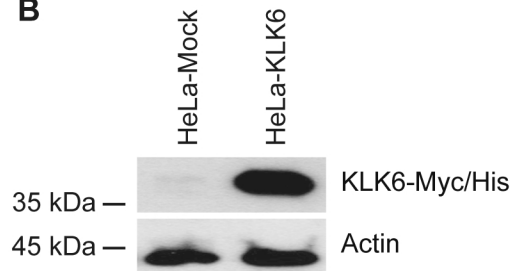**C**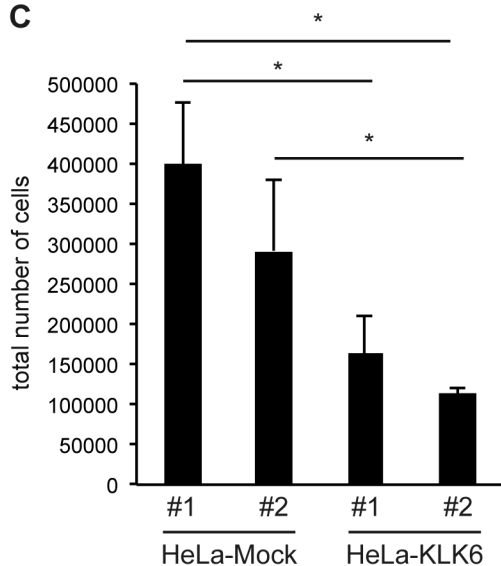**D**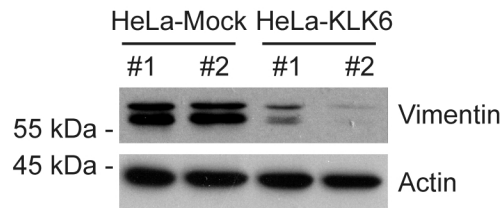

Supplement: Additional files 1: Figure S1. — Decreased tumor cell growth and Vimentin expression in HeLa cells with ectopic KLK6 expression. KLK6 transgene expression in stable HeLa clones was confirmed on transcript level by semi-quantitative RT-PCR (A) and on protein level by Western blot analysis (B). Detection of LMNB1 amplicons served as control for cDNA quality and quantity for semi-quantitative RT-PCR, while detection of β-Actin served as control for quantity and quality of protein lysates. (C) Differences in tumor cell growth between stable HeLa-Mock and HeLa-KLK6 clones were monitored by quantification of cell counts over a time period of one week. (D) Western blot analysis with whole cell lysate of HeLa-Mock and HeLa-KLK6 clones demonstrates reduced Vimentin protein levels in the presence of ectopic KLK6 expression. [file 12943_2015_381_MOESM1_ESM.pdf]

**A**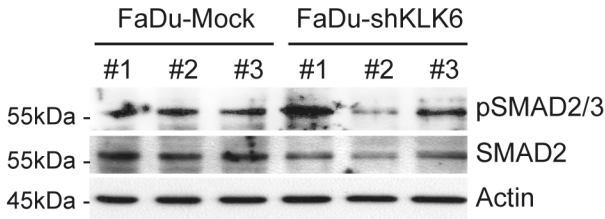**B**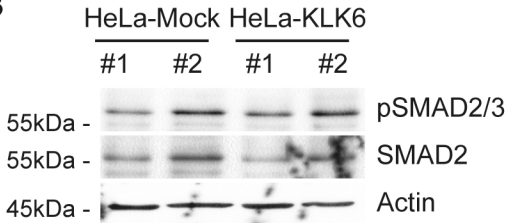

*Supplemental Figure S2\_Schrader et al., 2015*

Supplement: Additional files 2: Figure S2. — KLK6 expression has no impact on canonical TGFβ signaling. Western blot analysis with whole cell lysate of FaDu-Mock and FaDu-shKLK6 clones (A) or HeLa-Mock and HeLa-KLK6 clones (B) were conducted with anti-pSMAD2/3 or anti-SMAD2 antibodies. Detection of β-Actin served as control for quantity and quality of protein lysates. [file 12943_2015_381_MOESM2_ESM.pdf]

goat-anti-KLK6  
(AF2008; R&D)

rabbit-anti-KLK6  
(sc20264; Santa Cruz)

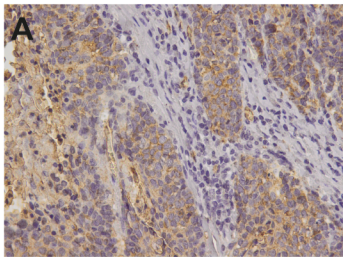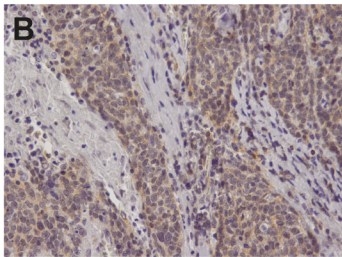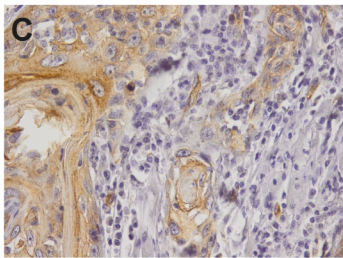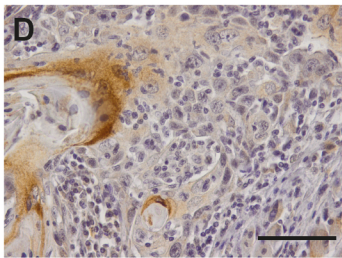

*Supplemental Figure S3\_Schrader et al., 2015*

Supplement: Additional files 3: Figure S3. — Confirmation of staining specificity by IHC staining with two independent anti-KLK6 antibodies. Representative pictures of an IHC staining (brown signal) with two independent anti-KLK6 antibodies (A and C, AF2008 from R&D; B and D, sc20264 from Santa Cruz) on serial TMA sections with primary HNSCC samples. Counterstaining with hematoxylin to visualize tissue architecture; black bar = 100 μm. [file 12943_2015_381_MOESM3_ESM.pdf]

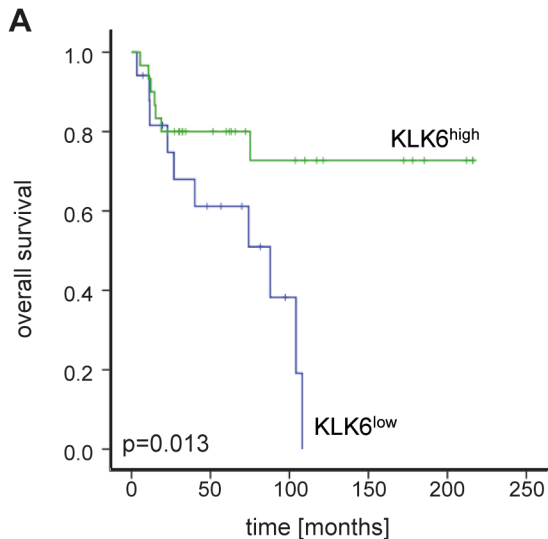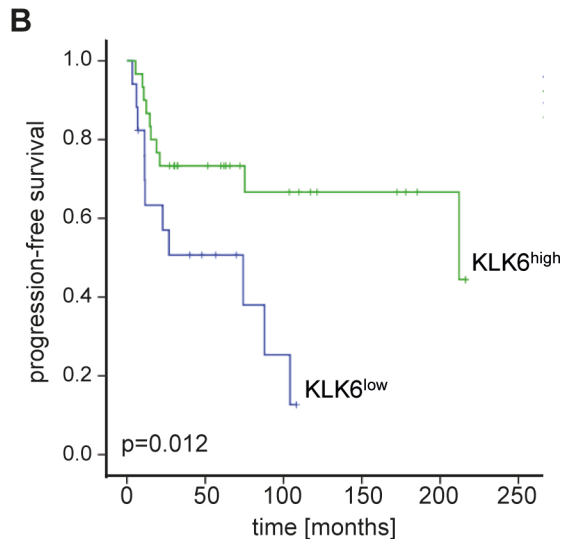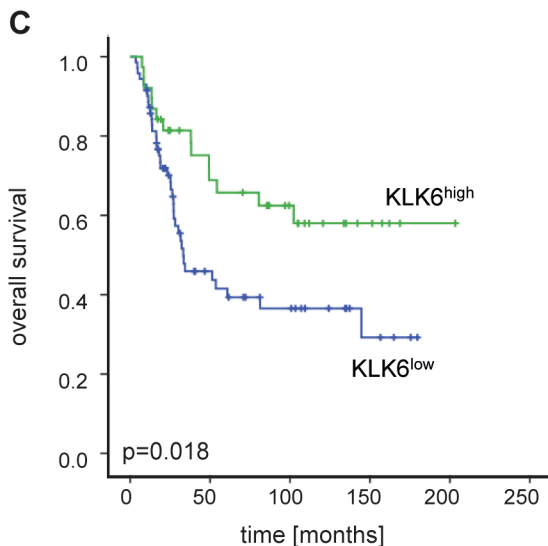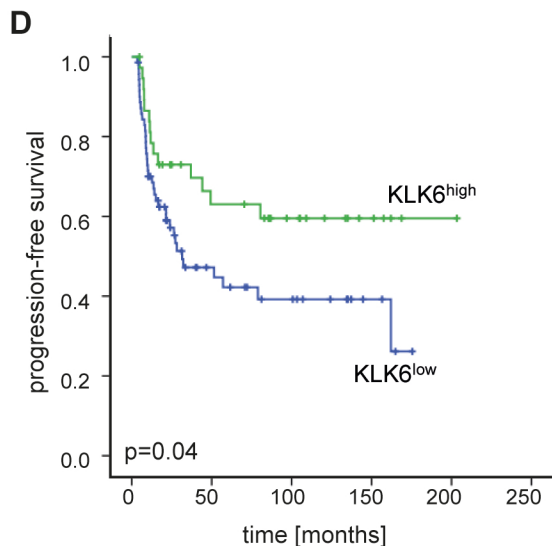

Supplement: Additional files 4: Figure S4. — KLK6 protein staining serves as risk factor for unfavorable progression-free and overall survival of LSCC and OPSCC patients. Association between KLK6 protein staining and overall survival (A and C) or progression-free survival (B and D) was assessed by univariate Kaplan-Meier analysis for the LSCC (A–B) and OPSCC patient cohort (C–D). Green line = subgroup with high KLK6 staining pattern and blue line = subgroup with low KLK6 staining pattern. [file 12943_2015_381_MOESM4_ESM.pdf]
