# Supplementary material for: Kallikrein-related peptidase 6 regulates epithelial-to-mesenchymal transition and serves as prognostic biomarker for head and neck squamous cell carcinoma patients
Source: Mol Cancer. 2015 May 20;14:107. doi: 10.1186/s12943-015-0381-6 (PMC4437453; doi:10.1186/s12943-015-0381-6)
Supplement: Additional files 5: Table S1. — List of primer sequences for RT-PCR analysisTable S2. List of primary (A) and secondary antibodies (B) Table S3. Correlation analysis for KLK6 protein expression and clinical and pathological features of the LSCC cohort. Table S4. Correlation analysis for KLK6 protein expression and clinical and pathological features of the OPSCC cohort. Table S5. Univariate Cox regression analysis of overall and progression-free survival for HNSCC patients of the combined cohort. [file 12943_2015_381_MOESM5_ESM.docx]

**Supplemental Table S1.** List of primer sequences for RT-PCR analysis

| **Gene Symbol** | | **Sequence** | **T_A_ (°C)** |
| --- | --- | --- | --- |
| *ACTB* | for | CCAACCGCGAGAAGATGA | 60 |
|  | rev | CCAGAGGCGTAGAGGGATAG |  |
| *CDH1* | for | AATCCCACCACGTACAAGGG | 60 |
|  | rev | GGTATTGGGGGCATCAGCAT |  |
| *CDH2* | for | CACCGTGGTCAAACCAATCG | 60 |
|  | rev | GGTGCTGAATTCCCTTGGCT |  |
| *FN1* | for | CTTTGGTGCAGCACAACTTC | 60 |
|  | rev | TCCTCCTCGAGTCTGAACCA |  |
| *KLK6* | for | CATGGCGGACCCTGCGACAAGAC | 64 |
|  | rev | AGGGGAAGGGGCTGGATGAGTT |  |
| *LMNB1* | for | CTGGAAATGTTTGCATCGAAGA | 60 |
|  | rev | GCCTCCCATTGGTGGATCC |  |
| *SNAI1* | for | GAGGCGGTGGCAGACTAG | 60 |
|  | rev | GACACATCGGTCAGACCAG |  |
| *SNAI2* | for | TGCGATGCCCAGTCTAGAAA | 60 |
|  | rev | AAAAGGCTTCTCCCCCGTGT |  |
| *TBP* | for | GAGCTGTGATGTGAAGTTTCC | 60 |
|  | rev | TCTGGGTTTGATCATTCTGTAG |  |
| *TWIST1* | for | GCCGGAGACCTAGATGTCATT | 60 |
|  | rev | TTTTAAAAGTGCGCCCCACG |  |
| *VIM* | for | CTCTGGCACGTCTTGACCTT | 60 |
|  | rev | TCCTGGATTTCCTCTTCGTG |  |
| *ZEB1* | for | AGCAGTGAAAGAGAAGGGAATGC | 60 |
|  | rev | GGTCCTCTTCAGGTGCCTCAG |  |
| *ZEB2* | for | TCCAGAAAAGCAGTTCCCTTC | 60 |
|  | rev | CACACTGATAGGGCTTCTCG |  |

**Supplemental Table S2.** List of primary (**A**) and secondary antibodies (**B**)

| A | Cat. No. | Clone & Species | Company | Application | Dilution |
| --- | --- | --- | --- | --- | --- |
| KLK6 | AF2008 | Polyclonal goat | R&D Systems | WB  IHC | 1:1000  1:200 |
| KLK6 | sc-20624 | H-60; polyclonal rabbit | Santa Cruz | IHC | 1:200 |
| E-cadherin | sc-8426 | G-10; monoclonal mouse | Santa Cruz | WB | 1:1000 |
| Vimentin | 61013 | VIM 3B4; monoclonal mouse | Progen | WB  IF | 1:1000  1:100 |
| SMAD2 | sc-6200 | S-20; polyclonal goat | Santa Cruz | WB | 1:2000 |
| Phospho-SMAD2/3 (Ser423/425) | sc-11769 | Polyclonal goat | Santa Cruz | WB | 1:200 |
| β-catenin | 610153 | Monoclonal mouse | BD Bioscience | IHC  IF | 1:200  1:200 |
| β-Actin | A5441 | AC-15; monoclonal mouse | Sigma-Aldrich | WB | 1:1000 |
| BrdU  Alexa555 | B35131 | MoBU-1; monoclonal mouse | Invitrogen | IF | 1:100 |
| Myc-Tag | sc-40 | 9E10; monoclonal mouse | Santa Cruz | WB | 1:1000 |

WB = Western blot, IHC = immunohistochemistry, IF = immunofluorescense

| B | Cat. No | Species | Company | Application | Dilution |
| --- | --- | --- | --- | --- | --- |
| anti-mouse-HRP | #7076 | Horse | Cell Signaling | WB | 1:10000-30000 |
| anti-goat-HRP | sc-2020 | Donkey | Santa Cruz | WB | 1:10000 |
| anti-goat-Biotin | BA-9500 | Horse | Vector Laboratories | IHC | 1:200 |
| anti-mouse-Alexa488 | A11001 | Goat | Life Technologies | IF | 1:200 |
| ImmPress® goat |  | Horse | Vector | IHC |  |
| ImmPress® mouse |  | Goat | Vector | IHC |  |

WB = Western blot, IHC = immunohistochemistry, IF = immunofluoreszense

**Supplemental Table S3.** Correlation analysis for KLK6 protein expression and clinical and pathological features of the LSCC cohort

|  |  | **KLK6^low^** | **KLK6^high^** | **p-value** |
| --- | --- | --- | --- | --- |
| Age [years] | <59.14 | 10 | 13 | 0.237 |
|  | ≥59.14 | 7 | 17 |  |
| Gender | male | 16 | 29 | 0.589 |
|  | female | 1 | 1 |  |
| Tumor size | T1/2 | 7 | 12 | 0.589 |
|  | T3/4 | 10 | 18 |  |
| Lymph node status | N0 | 12 | 20 | 0.524 |
|  | N+ | 5 | 10 |  |
| Distant metastasis | M0 | 16 | 28 | 0.608 |
|  | M+ | 1 | 1 |  |
|  | missing^1^ |  | 1 |  |
| Clinical stage | I/II | 6 | 8 | 0.382 |
|  | III/IV | 11 | 22 |  |
| Pathological grade | G1/2 | 6 | 22 | **0.038** |
|  | G3 | 8 | 7 |  |
|  | missing^1^ | 3 | 1 |  |
| Smoking status | non-smoker | 1 | 2 | 0.704 |
|  | smoker^2^ | 15 | 26 |  |
|  | missing^1^ | 1 | 2 |  |
| Alcohol consumption | non-drinker | 3 | 6 | 0.546 |
|  | drinker^3^ | 12 | 19 |  |
|  | missing^1^ | 2 | 5 |  |

^1^ data missing; ^2^ former and current smoker; ^3^ former and current drinker; significant p-values<0.05 are indicated in bold.

**Supplemental Table S4.** Correlation analysis for KLK6 protein expression and clinical and pathological features of the OPSCC cohort

|  |  | **KLK6^low^** | **KLK6^high^** | **p-value** |
| --- | --- | --- | --- | --- |
| Age [years] | <58.56 | 39 | 18 | 0.372 |
|  | ≥58.56 | 37 | 21 |  |
| Gender | male | 53 | 31 | 0.187 |
|  | female | 23 | 8 |  |
| Tumor size | T1/2 | 32 | 18 | 0.437 |
|  | T3/4 | 43 | 21 |  |
| Lymph node status | N0 | 15 | 7 | 0.501 |
|  | N+ | 60 | 32 |  |
| Distant metastasis | M0 | 71 | 39 | 0.277 |
|  | M+ | 3 | 0 |  |
| Clinical stage | I/II | 10 | 4 | 0.441 |
|  | III/IV | 65 | 35 |  |
| Pathological grade | G1/2 | 40 | 20 | 0.331 |
|  | G3 | 21 | 14 |  |
| Smoking status | non-smoker | 9 | 7 | 0.266 |
|  | smoker^1^ | 67 | 32 |  |
| Alcohol consumption | non-drinker | 5 | 3 | 0.550 |
|  | drinker^2^ | 71 | 36 |  |
| HPV status | non-related^3^ | 59 | 26 | **0.043** |
|  | related^4^ | 12 | 13 |  |

^1^ former and current smoker; ^2^ former and current drinker; ^3^ non-related = HPV-DNA and transcript negative or HPV-DNA positive but transcript negative; ^4^ related = HPV-DNA and transcript positive, according to Holzinger et al., 2013 Cancer Research; significant p-values<0.05 are indicated in bold.

**Supplemental Table S5. Univariate Cox regression analysis of overall and progression-free survival for HNSCC patients of the combined cohort**

| **Risk factor** | | **Overall Survival** | | **Progression-Free Survival** | |
| --- | --- | --- | --- | --- | --- |
|  | | **HR (95% CI)** | **p-value** | **HR (95% CI)** | **p-value** |
| KLK6 | |  |  |  |  |
|  | low vs. high^1^ | 2.552 (1.384-4.707) | **0.0027** | 1.727 (1.195-2.497) | **0.0036** |
| Age [years] | |  |  |  |  |
|  | <58.62^1^ vs. ≥58.62 | 1.350 (0.767-2.374) | 0.2977 | 1.122 (0.676-1.864) | 0.6554 |
| Gender | |  |  |  |  |
|  | male^1^ vs. female | 0.767 (0.372-1.582) | 0.4723 | 0.674 (0.341-1.332) | 0.2560 |
| Clinical stage | |  |  |  |  |
|  | I/II^1^ vs. III/IV | 2.036 (0.806-5.146) | 0.1328 | 1.592 (0.723-3.506) | 0.2481 |
| Pathological grade | |  |  |  |  |
|  | G1/2^1^ vs. G3 | 0.942 (0.518-1.714) | 0.8459 | 1.218 (0.719-2.062) | 0.4637 |
| smoking | |  |  |  |  |
|  | non-smoker^1^ vs. smoker^2^ | 1.633 (0.585-4.560) | 0.317 | 2.574 (0.931-7.116) | 0.0683 |
| Alcohol consumption | |  |  |  |  |
|  | non-drinker^1^ vs. drinker^3^ | 1.258 (0.498-3.178) | 0.6267 | 1.271 (0.545-2.961) | 0.5786 |

^1^variable set as reference; ^2^former and current smoker; ^3^former and current drinker. Significant p-values (<0.05) are indicated in bold. HR hazard ratio, CI confidence intervals.
